# Genetic Predisposition Impacts Clinical Changes in a Lifestyle Coaching Program

**DOI:** 10.1038/s41598-019-43058-0

**Published:** 2019-05-02

**Authors:** Niha Zubair, Matthew P. Conomos, Leroy Hood, Gilbert S. Omenn, Nathan D. Price, Bonnie J. Spring, Andrew T. Magis, Jennifer C. Lovejoy

**Affiliations:** 1 Arivale, Inc, Seattle, WA 98104 USA; 2 Department of Biostatistics, University of Washington, Seattle, WA USA; 3 Institute for Systems Biology, 401 Terry Ave N, Seattle, WA 98109 USA; 4 Providence St. Joseph Health, Seattle, Washington, USA; 5 Computational Medicine and Bioinformatics, Department of Human Genetics, Molecular Medicine and Genetics, School of Public Health, University of Michigan, Ann Arbor, MI USA; 6 Center for Behavior and Health, Institute for Public Health and Medicine, Feinberg School of Medicine, Northwestern University, Chicago, IL USA

**Keywords:** Quantitative trait, Genetic markers, Predictive markers, Preventive medicine

## Abstract

Both genetic and lifestyle factors contribute to an individual’s disease risk, suggesting a multi-omic approach is essential for personalized prevention. Studies have examined the effectiveness of lifestyle coaching on clinical outcomes, however, little is known about the impact of genetic predisposition on the response to lifestyle coaching. Here we report on the results of a real-world observational study in 2531 participants enrolled in a commercial “Scientific Wellness” program, which combines multi-omic data with personalized, telephonic lifestyle coaching. Specifically, we examined: 1) the impact of this program on 55 clinical markers and 2) the effect of genetic predisposition on these clinical changes. We identified sustained improvements in clinical markers related to cardiometabolic risk, inflammation, nutrition, and anthropometrics. Notably, improvements in HbA1c were akin to those observed in landmark trials. Furthermore, genetic markers were associated with longitudinal changes in clinical markers. For example, individuals with genetic predisposition for higher LDL-C had a lesser decrease in LDL-C on average than those with genetic predisposition for average LDL-C. Overall, these results suggest that a program combining multi-omic data with lifestyle coaching produces clinically meaningful improvements, and that genetic predisposition impacts clinical responses to lifestyle change.

## Introduction

Each individual has a unique and complex set of genetic, lifestyle, and environmental factors that impact clinical biomarkers and contribute to the manifestation of common conditions such as heart disease, diabetes, obesity, and hypertension. For this reason, a systems-based approach to quantifying wellness and detecting transitions to disease is well suited for prevention of chronic conditions common to modernized societies.

While there is strong scientific interest for using multi-omic data to prevent chronic diseases related to lifestyle and behavior, to date little value has been demonstrated for consumers or patients. For example, some studies have shown that simply receiving genetic information about one’s risk for chronic diseases does not lead to behavior change or actual risk reduction^1^, although more recent studies including an updated meta-analysis show modest behavior changes resulting from genetic information^2,3^. In addition, some scientists and physicians are understandably critical of providing genetic information in the absence of measuring the relevant clinical markers.

For some disease phenotypes, the relative contributions of genetics and lifestyle have been explored. One recent study found that a polygenic risk score and a lifestyle risk score had independent and additive effects on cardiovascular outcomes^4^. Because of the important effects of lifestyle on chronic disease risk, studies have also examined the effectiveness of health coaching on promoting clinical changes. Generally, these studies have found lifestyle coaching to be beneficial^5,6^. Furthermore, while there is some evidence that genetic predisposition has an impact on clinical response^7,8^, much less is known about the role of genetics in determining response to lifestyle change, supporting the need for further study.

To address these gaps, we developed a systems-based approach, “Scientific Wellness”, which combines genetic and clinical data with lifestyle coaching. For each individual, we generate personal, dense, dynamic data (PD3) clouds, which can aid in identifying unique actionable possibilities to optimize wellness and avoid disease. A critical component of Scientific Wellness is regular interaction with a registered dietitian or other allied healthcare provider for education and behavior modification. We previously published a pilot study that established the feasibility and potential clinical impact of this approach^9^.

Here we report on the results of a real-world observational study, using longitudinal blood, saliva, and anthropometric data gathered from 2531 participants in the commercial Scientific Wellness program (Arivale, Inc). We quantify the average change of each clinical marker for participants in their first year of the program, both in the population as a whole, and in clinically-relevant strata. For several clinical markers, we also examine the relationship between genetic predisposition and baseline values. Finally, we estimate the impact of genetic predisposition on changes in these clinical markers during the first year of the program.

## Results

The study cohort had a mean age of 48 years (SD = 12), approximately 60% were female, and 79% were White, 9% Asian, 4% Hispanic, and 2% Black. The number of participants with longitudinal observations for each clinical marker is shown in Table 1. The average time between measurements for blood lab data was 179 days (SD = 49), blood pressure 142 days (SD = 81), salivary cortisol 307 days (SD = 95), waist circumference 102 days (SD = 81), and weight 9 days (SD = 28).

**Table 1:** Clinical characteristics of the study population.

Clinical Marker^a^	N^b^	Mean (SD) Baseline^c^	Mean (SD) Latest^d^	% OOR Baseline^e^	% OOR Latest^f^	Δ OOR^g^
***Cardiovascular Markers***						
HDL-C, mg/dL	1809	63.2 (19.2)	63.0 (18.9)	7.9%	7.9%	0.0%
HDL particle number, μmol/L	1371	34.3 (6.0)	34.0 (5.9)	27.0%	28.4%	1.4%
Homocysteine, μmol/L	1572	10.3 (3.2)	9.5 (2.6)	7.8%	3.1%	−4.7%
LDL-C, mg/dL	1801	116.6 (33.5)	115.0 (32.0)	68.5%	67.6%	−0.9%
LDL particle number, nmol/L	1371	1315.2 (464.6)	1306.4 (459.2)	73.8%	73.8%	0.0%
LDL size, nm	1365	21.2 (0.5)	21.2 (0.5)	12.9%	10.5%	−2.3%
LDL small particle number, nmol/L	1371	459.3 (340.9)	439.5 (330.8)	33.5%	31.0%	−2.5%
Oxidized LDL cholesterol, U/L	1055	48.0 (15.9)	45.2 (14.5)	23.5%	16.6%	−6.9%
Total cholesterol, mg/dL	1809	200.1 (37.2)	197.2 (36.2)	47.5%	44.7%	−2.8%
Triglycerides, mg/dL	1809	101.6 (55.0)	95.7 (50.2)	13.5%	11.6%	−2.0%
***Insulin Resistance Markers***						
Adiponectin, μg/mL	1618	8.8 (6.9)	8.6 (6.1)	NA	NA	NA
Gamma-glutamyl transpeptidase (GGT), U/L	1946	23.3 (20.0)	21.2 (18.0)	4.2%	3.0%	−1.2%
Glucose, mg/dL	1990	91.8 (10.0)	91.8 (10.3)	15.9%	17.2%	1.3%
Hemoglobin A1c, %	1992	5.5 (0.4)	5.4 (0.4)	27.6%	22.6%	−4.9%
HOMA-IR	1990	2.4 (2.8)	2.2 (2.0)	NA	NA	NA
Insulin, μIU/mL	1989	10.1 (8.9)	9.3 (7.0)	5.6%	6.0%	0.4%
***Inflammatory Markers***						
hsCRP, mg/L	1584	2.6 (5.8)	2.2 (3.1)	23.3%	21.3%	−2.0%
IL-6, pg/mL	816	2.6 (22.2)	2.9 (23.8)	1.6%	1.5%	−0.1%
IL-8, pg/mL	817	14.3 (19.6)	12.9 (27.4)	2.4%	1.2%	−1.2%
TNF-alpha, pg/mL	815	1.1 (6.2)	1.7 (15.3)	2.0%	1.6%	−0.4%
Total neutrophils, %	2079	57.2 (8.9)	55.5 (8.8)	NA	NA	NA
***Stress Markers***						
DHEA, ng/mL	174	7.2 (5.8)	6.9 (6.2)	NA	NA	NA
Morning cortisol, ng/mL	335	7.6 (20.2)	7.4 (14.8)	23.9%	24.5%	0.6%
Noon cortisol, ng/mL	335	2.6 (2.2)	2.5 (1.5)	22.7%	25.7%	3.0%
Evening cortisol, ng/mL	335	1.7 (1.9)	1.6 (1.0)	23.6%	23.9%	0.3%
Night cortisol, ng/mL	334	1.1 (1.3)	1.0 (0.8)	32.9%	33.8%	0.9%
***Nutritional Markers***						
Arachidonic acid, % by wt	1578	10.6 (1.7)	10.8 (1.7)	NA	NA	NA
DHA, % by wt	1578	2.7 (0.8)	3.2 (0.8)	NA	NA	NA
DPA, % by wt	1578	1.1 (0.2)	1.3 (0.3)	NA	NA	NA
EPA, % by wt	1578	0.8 (0.4)	1.3 (0.7)	NA	NA	NA
Ferritin, ng/mL	1688	129.9 (134.6)	117.8 (111.3)	18.7%	12.6%	−6.1%
Hematocrit, %	2084	42.2 (3.6)	41.9 (3.2)	3.1%	1.4%	−1.7%
Hemoglobin, g/dL	2084	14 (1.3)	14.1 (1.2)	2.2%	1.2%	−1.0%
Linoleic acid, % by wt	1578	25.8 (2.7)	25.6 (2.6)	NA	NA	NA
Magnesium, mg/dL	996	2.0 (0.2)	2.1 (0.2)	2.2%	2.4%	0.2%
Mean corpuscular volume (MCV), fL	2084	90.4 (5.4)	90.2 (4.8)	10.6%	6.3%	−4.3%
Mercury, μg/L	1035	3.2 (4.6)	2.8 (3.3)	2.1%	0.7%	−1.4%
Methylmalonic acid (MMA), nmol/L	1436	171.2 (108.2)	152.8 (65.2)	3.3%	1.0%	−2.3%
Omega 3 index, %	1578	4.5 (1.3)	5.8 (1.6)	81.1%	42.5%	−38.6%
Vitamin D, ng/mL	2085	33.7 (14.7)	40.8 (14.3)	44.8%	20.4%	−24.4%
Zinc (red blood cell), μg/dL	559	1339 (255.2)	1387.8 (214)	15.9%	16.6%	0.7%
***Blood Pressure & Anthropometrics***						
Diastolic, mmHg	1804	76.7 (10.3)	74.3 (10.1)	40.2%	29.5%	−10.7%
Systolic, mmHg	1804	122.7 (14.7)	120.8 (14.1)	56.2%	50.2%	−6.0%
Body mass index (BMI), kg/m^2^	2355	28.2 (6.4)	27.6 (6.1)	64.5%	60.1%	−4.4%
Waist circumference, inches	1325	37.3 (7.4)	36.1 (7.7)	41.7%	32.1%	−9.7%
Weight, lbs	2355	181.9 (44.8)	178.1 (42.7)	64.5%	60.1%	−4.4%

^a^Clinical marker of interest followed by unit.

^b^Number of individuals included in longitudinal analysis of corresponding clinical marker.

^c^Mean and standard deviation of corresponding clinical marker at baseline measurement.

^d^Mean and standard deviation of corresponding clinical marker at latest measurement.

^e^% of individuals with out of range values of corresponding clinical marker at baseline measurement; NA if either no out of range definition or less than 50 individuals out of range.

^f^% of individuals with out of range values of corresponding clinical marker at latest measurement; NA see above.

^g^Change in out of range % from baseline to latest measurement; NA see above.

N: number of individuals; NA: not applicable; OOR: out of range; △ OOR: change in out of range from baseline to latest measurement; SD: standard deviation.

Liver and Kidney markers are shown in Supplementary Table 1.

In total, 55 clinical markers were analyzed. At baseline and latest measurements, population means, SDs, and % of participants out of range (OOR) were recorded (Table 1 and Supplementary Table 1).

### Longitudinal Changes

**Figure 1:**
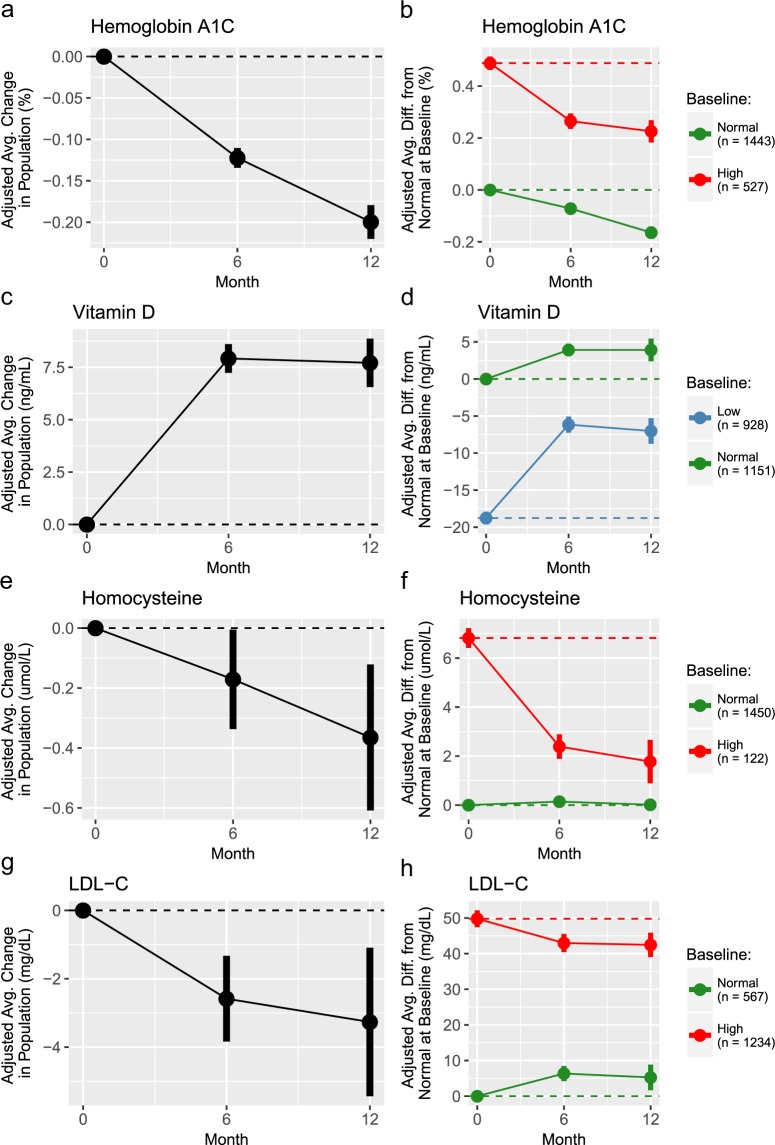
Longitudinal changes for select clinical markers. Panels a,c,e, and g: Adjusted changes for the average participant in the entire study population. Panels b,d,f,h: Adjusted average differences from the ‘normal at baseline’ strata at baseline for each baseline strata over time in the program. In panels, the points represent the estimates from the GLMMs, and the vertical bars show the 95% confidence intervals. The solid lines connecting the points visually shows the trajectories over time and the dashed horizontal lines show the starting value of the entire population or corresponding strata.

We estimated 6- and 12-month changes for the average participant, adjusted for confounding effects; we refer to these as “adjusted changes” throughout. These adjusted changes were estimated for the entire participant population, as well as for strata defined by baseline reference ranges (‘normal at baseline’, ‘low at baseline’, and ‘high at baseline’) when available and with sufficient sample size (Fig. 1, Table 2 and Supplementary Table 2).

**Table 2:** Longitudinal changes from generalized linear mixed model analyses stratified by baseline range.

Clinical Marker^a^	Baseline Strata^b^	Range^c^	N^d^	Adj 6 mo Δ^e^	Adj 12 mo Δ^f^
***Cardiovascular Markers***					
HDL-C, mg/dL	All		1809	0.23 (−0.27, 0.73)	−0.40 (−1.28, 0.48)
	Low	< = 39.00	143	**2.75 (1.01, 4.50)**	2.97 (−0.42, 6.35)
	Normal	>39.00	1666	−0.04 (−0.56, 0.48)	−0.73 (−1.63, 0.18)
HDL particle number, μmol/L	All		1371	−**0.45 (**−**0.72**, −**0.18)**	−**0.48 (**−**0.93**, −**0.03)**
	Low	<30.50	370	**1.39 (0.88, 1.89)**	**1.75 (0.87, 2.62)**
	Normal	> = 30.50	1001	−**1.13 (**−**1.44**, −**0.82)**	−**1.33 (**−**1.84**, −**0.82)**
Homocysteine, μmol/L	All		1572	−**0.17 (**−**0.34**, −**0.01)**	−**0.36 (**−**0.61**, −**0.12)**
	Normal	< = 15.00	1450	0.14 (−0.02, 0.30)	0.01 (−0.22, 0.25)
	High	>15.00	122	−**4.43 (**−**4.96**, −**3.91)**	−**5.04 (**−**5.93**, −**4.16)**
LDL-C, mg/dL	All		1801	−**2.58 (**−**3.84**, −**1.33)**	−**3.26 (**−**5.44**, −**1.09)**
	Normal	< = 99.00	567	**6.36 (4.23, 8.48)**	**5.26 (1.68, 8.85)**
	High	>99.00	1234	−**6.81 (**−**8.27**, −**5.36)**	−**7.32 (**−**9.95**, −**4.70)**
LDL particle number, nmol/L	All		1371	−0.42 (−20.83, 19.99)	−13.20 (−46.81, 20.42)
	Normal	<1000.00	359	**80.02 (40.97, 119.07)**	**63.89 (2.95, 124.83)**
	High	> = 1000.00	1012	−**29.70 (**−**53.11**, −**6.29)**	−**40.76 (**−**80.46**, −**1.07)**
LDL size, nm	All		1365	0.00 (−0.03, 0.02)	**0.07 (0.03, 0.11)**
	Low	< = 20.50	176	**0.32 (0.25, 0.39)**	**0.41 (0.30, 0.52)**
	Normal	>20.50	1189	−**0.05 (**−**0.08**, −**0.02)**	0.02 (−0.03, 0.07)
LDL small particle number, nmol/L	All		1371	−0.89 (−9.87, 8.09)	−**15.09 (**−**28.95**, −**1.24)**
	Normal	< = 527.00	912	**9.67 (0.63, 18.70)**	−6.51 (−20.09, 7.07)
	High	>527.00	459	−**127.87 (**−**174.16**, −**81.57)**	−**158.36 (**−**225.66**, −**91.07)**
Oxidized LDL cholesterol, U/L	All		1055	−**2.33 (**−**3.49**, −**1.17)**	−**5.52 (**−**7.09**, −**3.95)**
	Normal	<60.00	807	**1.73 (0.51, 2.95)**	−1.16 (−2.79, 0.48)
	High	> = 60.00	248	−**15.38 (**−**17.31**, −**13.45)**	−**18.32 (**−**21.56**, −**15.09)**
Total cholesterol, mg/dL	All		1809	−**3.40 (**−**4.87**, −**1.93)**	−**4.91 (**−**7.40**, −**2.42)**
	Normal	> = 100.00 and<200.00	950	**4.22 (2.30, 6.14)**	**3.78 (0.51, 7.05)**
	High	> = 200.00	859	−**11.96 (**−**13.98**, −**9.95)**	−**14.83 (**−**18.40**, −**11.27)**
Triglycerides, mg/dL	All		1809	−**3.69 (**−**5.23**, −**2.15)**	−**4.71 (**−**7.14**, −**2.28)**
	Normal	< = 149.00	1564	−1.32 (−2.79, 0.15)	−**2.77 (**−**5.08**, −**0.46)**
	High	>149.00	245	−**53.60 (**−**61.34**, −**45.86)**	−**46.26 (**−**58.55**, −**33.96)**
***Insulin Resistance Markers***					
Adiponectin, μg/mL	All		1618	0.01 (−0.11, 0.12)	0.02 (−0.15, 0.18)
Gamma-glutamyl transpeptidase (GGT), U/L	All		1946	−**0.51 (**−**0.78**, −**0.24)**	−**0.79 (**−**1.21**, −**0.37)**
	Normal	< = 65.0 (M);< = 60.0 (F)	1864	−**0.43 (**−**0.68**, −**0.18)**	−**0.67 (**−**1.07**, −**0.28)**
	High	>65.0 (M); >60.0 (F)	82	−**29.99 (**−**35.75**, −**24.24)**	−**38.17 (**−**44.8**, −**31.54)**
Glucose, mg/dL	All		1990	0.13 (−0.28, 0.54)	0.56 (−0.09, 1.22)
	Normal	> = 65.00 and< = 99.00	1673	**0.94 (0.53, 1.36)**	**1.38 (0.70, 2.05)**
	High	>99.00	317	−**5.67 (**−**6.77**, −**4.57)**	−**5.37 (**−**7.02**, −**3.72)**
Hemoglobin A1c, %	All		1992	−**0.12 (**−**0.13**, −**0.11)**	−**0.20 (**−**0.22**, −**0.18)**
	Normal	> = 4.80 and < = 5.60	1443	−**0.07 (**−**0.09**, −**0.06)**	−**0.16 (**−**0.19**, −**0.14)**
	High	>5.60	527	−**0.22 (**−**0.24**, −**0.20)**	−**0.26 (**−**0.30**, −**0.23)**
HOMA−IR	All		1990	0.02 (−0.03, 0.06)	0.02 (−0.05, 0.08)
Insulin, μIU/mL	All		1989	0.06 (−0.11, 0.23)	0.06 (−0.22, 0.34)
	Normal	> = 2.60 and < = 24.90	1878	0.04 (−0.13, 0.21)	−0.02 (−0.30, 0.26)
	High	>24.90	81	−**10.99 (**−**14.2**, −**7.77)**	−**8.17 (**−**13.09**, −**3.25)**
***Inflammatory Markers***					
hsCRP, mg/L	All		1584	**0.06 (0.02, 0.09)**	0.03 (−0.02, 0.09)
	Normal	< = 3.00	1215	**0.10 (0.06, 0.15)**	0.06 (0.00, 0.12)
	High	>3.00	369	−**1.75 (**−**2.34**, −**1.17)**	−**2.88 (**−**3.64**, −**2.13)**
IL-6, pg/mL	All		816	**0.14 (0.02, 0.26)**	−0.13 (−0.34, 0.09)
IL-8, pg/mL	All		817	−0.87 (−1.85, 0.11)	−**2.09 (**−**3.86**, −**0.33)**
TNF-alpha, pg/mL	All		815	**0.07 (0.00, 0.13)**	**0.22 (0.09, 0.35)**
Total neutrophils, %	All		2079	−**0.69 (**−**1.06**, −**0.32)**	−**0.93 (**−**1.53**, −**0.33)**
***Stress Markers***					
DHEA, ng/mL	All		174	0.40 (−0.46, 1.26)	−0.93 (−1.93, 0.06)
Morning cortisol, ng/mL	All		335	−0.57 (−1.83, 0.69)	0.13 (−1.15, 1.40)
	Low	<3.70	52	**2.21 (0.47, 3.95)**	**2.97 (1.57, 4.37)**
	Normal	> = 3.70 and < = 9.50	255	−**1.14 (**−**1.92**, −**0.37)**	−0.06 (−0.78, 0.67)
Noon cortisol, ng/mL	All		335	−0.26 (−0.72, 0.21)	−0.16 (−0.43, 0.11)
	Normal	> = 1.20 and < = 3.00	259	0.17 (−0.32, 0.65)	0.02 (−0.22, 0.26)
	High	>3.00	60	−**1.97 (**−**2.92**, −**1.02)**	−**2.35 (**−**3.3**, −**1.39)**
Evening cortisol, ng/mL	All		335	−0.27 (−0.56, 0.02)	−0.04 (−0.23, 0.14)
	Normal	> = 0.60 and < = 1.90	256	0.04 (−0.21, 0.30)	**0.19 (0.03, 0.35)**
	High	>1.90	69	−**1.65 (**−**2.31**, −**0.99)**	−**1.99 (**−**2.54**, −**1.44)**
Night cortisol, ng/mL	All		334	−0.17 (−0.41, 0.07)	−0.09 (−0.23, 0.04)
	Normal	> = 0.40 and< = 1.00	224	0.04 (−0.16, 0.24)	**0.15 (0.02, 0.28)**
	High	>1.00	104	−0.44 (−0.93, 0.06)	−**1.06 (**−**1.36**, −**0.75)**
***Nutritional Markers***					
Arachidonic acid, % by wt	All		1578	0.05 (−0.04, 0.15)	**0.32 (0.16, 0.48)**
DHA, % by wt	All		1578	**0.67 (0.62, 0.71)**	**0.53 (0.45, 0.61)**
DPA, % by wt	All		1578	**0.21 (0.19, 0.22)**	**0.21 (0.19, 0.24)**
EPA, % by wt	All		1578	**0.65 (0.61, 0.69)**	**0.59 (0.51, 0.66)**
Ferritin, ng/mL	All		1688	−1.30 (−2.88, 0.29)	−**7.88 (**−**10.67**, −**5.09)**
	Low	<30.00 (M);<15.00 (F)	109	**8.36 (6.43, 10.29)**	**9.56 (4.38, 14.75)**
	Normal	> = 30.00 and< = 400.00 (M); > = 15.00 and < = 150.00 (F)	1372	−1.39 (−2.93, 0.14)	−**4.41 (**−**6.89**, −**1.93)**
	High	>400.00 (M); >150.00 (F)	207	−**38.62 (**−**53.49**, −**23.75)**	−**59.81 (**−**83.31**, −**36.31)**
Hematocrit, %	All		2084	−0.02 (−0.14, 0.09)	−0.13 (−0.33, 0.06)
Hemoglobin, g/dL	All		2084	−0.01 (−0.05, 0.03)	−0.05 (−0.11, 0.02)
Linoleic acid, % by wt	All		1578	−**0.18 (**−**0.32**, −**0.05)**	−0.17 (−0.39, 0.05)
Magnesium, mg/dL	All		996	0.01 (0.00, 0.03)	0.01 (0.00, 0.03)
Mean corpuscular volume (MCV), fL	All		2084	**0.39 (0.25, 0.52)**	**0.44 (0.21, 0.67)**
	Low	<79.00	52	**4.55 (3.76, 5.34)**	**5.20 (3.57, 6.84)**
	Normal	> = 79.00 and< = 97.00	1863	**0.32 (0.19, 0.46)**	**0.38 (0.15, 0.61)**
	High	>97.00	169	−**1.06 (**−**1.51**, −**0.61)**	−**1.53 (**−**2.39**, −**0.67)**
Mercury, μg/L	All		1035	0.06 (−0.05, 0.17)	−0.04 (−0.14, 0.05)
Methylmalonic acid (MMA), nmol/L	All		1436	−**4.57 (**−**8.17**, −**0.96)**	−**12.31 (**−**16.83**, −**7.79)**
Omega 3 index, %	All		1578	**1.52 (1.43, 1.61)**	**1.30 (1.14, 1.45)**
	Low	< = 5.40	1279	**1.69 (1.60, 1.79)**	**1.53 (1.34, 1.71)**
	Normal	>5.40	299	**0.80 (0.61, 0.99)**	0.24 (−0.15, 0.62)
Vitamin D, ng/mL	All		2085	**7.92 (7.24, 8.60)**	**7.71 (6.56, 8.87)**
	Low	<30	928	**12.60 (11.67, 13.54)**	**11.74 (10.14, 13.34)**
	Normal	> = 30.00 and < = 100.00	1151	**3.91 (3.06, 4.77)**	**3.92 (2.39, 5.44)**
Zinc (red blood cell), μg/dL	All		559	**33.76 (10.45, 57.08)**	86.05 (−4.93, 177.03)
	Normal	> = 822.00 and< = 1571.00	470	**74.01 (50.12, 97.89)**	**125.40 (36.47, 214.34)**
	High	>1571.00	85	−**173.44 (**−**227.95**, −**118.93)**	78.02 (−369.45, 525.49)
***Blood Pressure & Anthropometrics***					
Diastolic, mmHg	All		1804	−**1.77 (**−**2.26**, −**1.28)**	−**1.23 (**−**1.98**, −**0.47)**
	Normal	<80.00	1078	**0.71 (0.11, 1.31)**	0.47 (−0.57, 1.50)
	High	> = 80.00	726	−**5.56 (**−**6.26**, −**4.85)**	−**3.93 (**−**5.06**, −**2.80)**
Systolic, mmHg	All		1804	−**2.29 (**−**2.99**, −**1.58)**	−**1.34 (**−**2.40**, −**0.27)**
	Normal	<120.00	790	**2.40 (1.38, 3.42)**	**1.71 (0.09, 3.33)**
	High	> = 120.00	1014	−**5.38 (**−**6.25**, −**4.51)**	−**3.23 (**−**4.60**, −**1.85)**
Body mass index (BMI), kg/m^2^	All		2355	−**0.65 (**−**0.70**, −**0.61)**	−**0.52 (**−**0.61**, −**0.43)**
	Normal	> = 18.50 and<25.00	836	−**0.22 (**−**0.30**, −**0.14)**	0.03 (−0.12, 0.17)
	Overweight	> = 25.00 and<30.00	781	−**0.58 (**−**0.66**, −**0.51)**	−**0.48 (**−**0.63**, −**0.33)**
	Obese	> = 30.00	723	−**1.16 (**−**1.24**, −**1.08)**	−**1.17 (**−**1.33**, −**1.02)**
Waist circumference, inches	All		1325	−**1.18 (**−**1.37**, −**0.99)**	−**1.59 (**−**1.93**, −**1.25)**
	Normal	< = 40.00 (M);< = 35.00 (F)	772	−**0.47 (**−**0.72**, −**0.22)**	−**0.76 (**−**1.20**, −**0.32)**
	High	>40.00 (M);>35.00 (F)	553	−**2.08 (**−**2.35**, −**1.81)**	−**2.67 (**−**3.14**, −**2.20)**
Weight, lbs	All		2355	−**4.22 (**−**4.51**, −**3.92)**	−**3.38 (**−**3.96**, −**2.80)**
	Normal	BMI> = 18.50 and<25.00	836	−**1.48 (**−**1.97**, −**0.99)**	0.03 (−0.92, 0.99)
	Overweight	BMI> = 25.00 and<30.00	781	−**3.78 (**−**4.27**, −**3.28)**	−**3.25 (**−**4.21**, −**2.28)**
	Obese	BMI> = 30.00	723	−**7.40 (**−**7.90**, −**6.89)**	−**7.40 (**−**8.39**, −**6.41)**

^a^Clinical marker of interest followed by unit.

^b^Strata as defined by baseline measurement of corresponding clinical marker; All: all participants regardless of strata; Low: those low at baseline; Normal: those normal at baseline, High: those high at baseline. Strata only shown for markers with defined ranges and with 50 or more individuals.

^c^Range used to define strata for corresponding clinical marker; sex-specific where indicated.

^d^Number of individuals included in longitudinal analysis of corresponding clinical marker and strata.

^e^Estimated 6 month changes and 95% CIs for the average participant, adjusted for confounding effects.

^f^Estimated 12 month changes and 95% CIs for the average participant, adjusted for confounding effects.

Adj 6 mo Δ: adjusted 6 month change; Adj 12 mo Δ: adjusted 12 month change; F: female; M: male; N: number of individuals.

Bolded values indicate p<0.05.

Liver and Kidney markers are shown in Supplementary Table 2, which also contains p-values and adjusted p-values.

There was evidence of sustained improvements in clinical markers related to cardiometabolic risk, inflammation, nutrition, and anthropometrics. Several clinical markers, including triglycerides, gamma-glutamyl transpeptidase (GGT), hemoglobin A1c (HbA1c), omega-3 index, vitamin-D, waist circumference, and weight, had improvements in the entire population as well as in each baseline strata. Some of these clinical markers, such as HbA1c (Fig. 1a,b), had improvements from baseline to 6 months as well as from 6 to 12 months, while others, such as Vitamin D (Fig. 1c,d), had improvements from baseline at both 6 and 12 months, but remained stable between 6 and 12 months.

Other clinical markers, such as HDL-C, homocysteine, and insulin, showed improvements in the baseline OOR strata, but showed no evidence of change in the baseline normal strata. When considering the entire population, HDL-C showed no evidence of change, while homocysteine (Fig. 1e,f), showed improvements.

Lastly, markers such as LDL-C, glucose, hs-CRP, and diastolic and systolic blood pressure, had improvements in the baseline OOR strata, but had worsening in the normal strata. This pattern of changes may be indicative of regression to the mean effects arising due to measurement variability, along with using strata defined by baseline observations of the outcome variable^10^. Regression to the mean leads to biased overestimates of changes in strata analyses; however, it does not bias estimates of changes in the entire population, for which some of these markers showed improvements, such as LDL-C (Fig. 1g,h) and diastolic and systolic blood pressure.

### Phenotypic Variation in Baseline Measures Explained by Genetic Markers

Associations were replicated between 11 of 13 genetic markers tested and the baseline measurements of clinical markers with which they were expected to be correlated. The most informative polygenic scores (PGSs) were for low-density lipoprotein cholesterol or LDL-C (11.1% variation explained), total cholesterol (8.7%), high-density lipoprotein cholesterol or HDL-C (6.9%), and triglycerides (3.9%). Compared to participants with LDL-C PGS in the second or third quartile (Q2/Q3) of the population (i.e. genetic predisposition for average LDL-C), participants with LDL-C PGS in the first quartile (Q1) of the population (genetic predisposition for lower LDL-C) had adjusted baseline LDL-C that was 15.7 mg/dL lower on average. Conversely, participants with LDL-C PGS in the fourth quartile (Q4) of the population (genetic predisposition for higher LDL-C) had adjusted baseline LDL-C that was 13.7 mg/dL higher on average.

The most informative single nucleotide polymorphisms (SNPs) were rs174537 (13.8% variation explained for arachidonic acid and 1.8% for EPA) and rs4588 (1.5% variation explained for vitamin D). Compared to participants with the GG genotype at rs4588, participants with the GT and TT genotypes had adjusted baseline vitamin D that was 1.8 ng/mL lower and 6.0 ng/mL lower on average, respectively. The percent of variation explained for PGS and SNPs used in this study were comparable to the original studies^11,12^. The partial r^2^ and estimated effect sizes for each clinical-genetic marker pair tested are presented in Table 3 and Supplementary Table 3.

**Table 3:** Effects of genetics on baseline levels and on longitudinal changes of clinical markers.

Clinical Marker^a^	Genetic Feature^b^	Baseline % Variation Explained^c^	Genetic strata^d^	Difference at Baseline^e^	Difference in Longitudinal Δ^f^
LDL-C, mg/dL	LDL-C PGS	11.1%	Q1	**−15.74 (−18.80, −12.59)**	**−3.82 (−5.92, −1.72)**
			Q2/Q3	Ref	Ref
			Q4	**13.69 (10.16, 17.22)**	−0.91 (−3.00, 1.18)
Total Cholesterol, mg/dL	Total Cholesterol PGS	8.7%	Q1	**−13.82 (−17.42, −10.22)**	**−4.32 (−6.77, −1.88)**
			Q2/Q3	Ref	Ref
			Q4	**15.79 (11.95, 19.64)**	−0.61 (−3.04, 1.81)
HDL-C, mg/dL	HDL-C PGS	6.9%	Q1	**−6.02 (−7.75, −4.28)**	−0.58 (−1.47, 0.32)
			Q2/Q3	Ref	Ref
			Q4	**6.67 (4.79, 8.55)**	**1.25 (0.38, 2.12)**
Triglycerides, mg/dL	Triglycerides PGS	3.9%	Q1	**−13.44 (−18.34, −8.55)**	−1.96 (−5.44, 1.52)
			Q2/Q3	Ref	Ref
			Q4	**15.76 (9.62, 21.90)**	**5.14 (1.68, 8.60)**
Waist circumference (Females), inches	Waist circumference PGS (Females)	3.2%	Q1	−0.98 (−2.00, 0.04)	−0.37 (−0.80, 0.05)
			Q2/Q3	Ref	Ref
			Q4	**1.98 (0.86, 3.10)**	−0.29 (−0.71, 0.13)
Waist circumference (Males), inches	Waist circumference PGS (Males)	1.8%	Q1	−0.47 (−1.45, 0.51)	0.18 (−0.26, 0.62)
			Q2/Q3	Ref	Ref
			Q4	**1.41 (0.34, 2.47)**	0.04 (−0.39, 0.46)
Body mass index (BMI Females), kg/m^2^	BMI PGS (Females)	2.7%	Q1	**−1.39 (−2.19, −0.58)**	0.04 (−0.10, 0.19)
			Q2/Q3	Ref	Ref
			Q4	**1.71 (0.84, 2.58)**	0.09 (−0.05, 0.23)
Body mass index (BMI Males), kg/m^2^	BMI PGS (Males)	1.7%	Q1	−0.22 (−0.90, 0.47)	0.02 (−0.14, 0.17)
			Q2/Q3	Ref	Ref
			Q4	**1.33 (0.54, 2.13)**	0.09 (−0.06, 0.25)
Homocysteine, μmol/L	Homocysteine PGS	0.7%	Low	Ref	Ref
			Moderate	0.01 (−0.28, 0.30)	−0.19 (−0.39, 0.00)
			High	**0.73 (0.20, 1.26)**	−0.03 (−0.34, 0.28)
Arachidonic acid, % by wt	rs174537	13.8%	TT	Ref	Ref
			GT	**0.9 (0.65, 1.14)**	**0.32 (0.12, 0.52)**
			GG	**1.83 (1.59, 2.08)**	**0.63 (0.41, 0.85)**
EPA, % by wt	rs174537	1.8%	TT	Ref	Ref
			GT	**0.11 (0.05, 0.17)**	0.05 (−0.04, 0.15)
			GG	**0.17 (0.11, 0.23)**	0.07 (−0.02, 0.17)
DPA, % by wt	rs174537	1.8%	TT	Ref	Ref
			GT	**0.05 (0.01, 0.09)**	0.02 (−0.01, 0.05)
			GG	**0.09 (0.05, 0.12)**	0.01 (−0.02, 0.05)
Vitamin D, ng/mL	rs4588	1.5%	GG	Ref	Ref
			GT	**−1.81 (−2.97, −0.64)**	0.11 (−0.79, 1.00)
			TT	**−6.01 (−8.17, −3.86)**	−1.68 (−3.43, 0.06)
hsCRP, mg/L	rs1205	0.5%	TT	Ref	Ref
			CT	0.55 (−0.04, 1.14)	0.05 (−0.31, 0.41)
			CC	**0.89 (0.30, 1.47)**	0.21 (−0.15, 0.58)
Systolic, mmHg	rs699	0.1%	AA	Ref	Ref
			AG	−0.61 (−2.11, 0.88)	0.12 (−0.91, 1.15)
			GG	−0.85 (−2.79, 1.09)	0.06 (−1.26, 1.38)
Diastolic, mmHg	rs699	0.0%	AA	Ref	Ref
			AG	−0.13 (−1.17, 0.91)	−0.24 (−0.97, 0.49)
			GG	0.24 (−1.16, 1.65)	−0.75 (−1.68, 0.18)

^a^Clinical marker of interest followed by unit.

^b^Genetic feature used to measure genetic predisposition for corresponding clinical marker; polygenic scores (PGS) or single nucleotide polymorphisms as indicated.

^c^% variation explained of baseline levels of corresponding clinical marker by genetic feature.

^d^Strata examined, defined by genetic feature; quartile (Q) or genotype as indicated.

^e^Estimated difference in baseline levels of corresponding clinical marker compared to reference genetic strata.

^f^Estimated difference in longitudinal change of corresponding clinical marker compared to reference genetic strata.

Q1/2/3/4: quartile 1/2/3/4; Ref: reference genetic strata.

Bolded values indicate p<0.05.

See Supplementary Table 3 for p-values and adjusted p-values.

### Effect of Genetics on Longitudinal Changes

The SNPs with the strongest genetic effects on longitudinal changes of associated clinical markers were the same SNPs that were most informative for baseline levels of those clinical markers (Table 3 and Supplementary Table 3). The G allele of rs174537 was additively associated with higher baseline levels of both arachidonic acid and EPA among participants in the program. Interestingly, having more copies of the G allele was associated with a greater increase of arachidonic acid through the course of the program (0.3% by wt. for GT vs TT, and 0.6% by wt. for GG vs. TT), but no difference in change of EPA.

We found similar longitudinal effects on differential change of clinical markers for the lipid PGSs (Table 3 and Supplementary Table 3). Adjusting for baseline LDL-C, those with an LDL-C PGS in Q1 (predisposed to lower/better LDL-C levels) had a 3.8 mg/dL greater decrease in LDL-C on average than those with an LDL-C PGS in Q2 or Q3 after the same amount of time in the program (Supplementary Figure 1). Adjusting for baseline total cholesterol, those with a total cholesterol PGS in Q1 (predisposed to lower/better total cholesterol levels) had a 4.3 mg/dL greater decrease in total cholesterol on average than those with a total cholesterol PGS in Q2 or Q3 after the same amount of time in the program. Adjusting for baseline HDL-C, those with an HDL-C PGS in Q4 (predisposed to higher/better HDL-C levels) had a 1.3 mg/dL greater increase in HDL-C on average than those with an HDL-C PGS in Q2 or Q3 after the same amount of time in the program. Lastly, adjusting for baseline triglycerides, those with a triglycerides PGS in Q4 (predisposed to higher/worse triglycerides levels) had a 5.1 mg/dL lesser decrease in triglycerides on average than those with a triglycerides PGS in Q2 or Q3 after the same amount of time in the program.

## Discussion

This study extends the results of previous studies^9,13^ and supports the importance of a Scientific Wellness approach, combining multi-omic data and personalized lifestyle coaching in a real-world setting. First, participants saw notable improvements in multiple clinical markers related to health, many of which were observed in the entire population, not just in those who began with out of range values. Second, previously reported associations between genetic markers and select clinical markers were replicated. Most intriguingly, some genetic markers were found to be associated with differences in the longitudinal changes in response to this lifestyle coaching program. These results suggest that certain genetic predispositions have an effect on the magnitude of change in clinical markers achieved through this program.

Some clinical improvements observed in this real-world study were comparable to improvements seen in diet and lifestyle randomized controlled trials (RCTs). For example, we found an adjusted average decrease in HbA1c of about 0.20% at 12 months in the entire study population. Among those participants with elevated baseline HbA1c, the adjusted average decrease was 0.26% at 12 months. The Diabetes Prevention Program, a RCT comparing intensive lifestyle intervention vs. metformin/standard care, saw slightly less than a 0.1% decrease in HbA1c at 12 months for those in the lifestyle intervention arm^14^. According to a meta-analysis, a 0.16% improvement in HbA1c in prediabetes is associated with at least a 1% reduction in the annualized incidence of diabetes, or an estimated 880,000 fewer cases of diabetes per year in the U.S^15,16^. These findings highlight that a program founded in systems biology and behavioral theory, and using scalable telephonic coaching, can provide improvements in glycemic health that compare to those seen in landmark clinical trials, and could have a meaningful impact on public health.

The estimated effect sizes of many of the genetics used in this study on baseline lab markers were clinically meaningful. For example, the difference in average adjusted baseline LDL-C was 29.4 mg/dL between LDL-C PGS Q1 and Q4. In previous studies, a reduction of similar magnitude in LDL-C (38.7 mg/dL) was found to be associated with a 23% reduction in relative risk of major vascular events^17^. Additionally, the LDL-C PGS was associated with differences in longitudinal changes of LDL-C, controlling for baseline values and time in the program. On average, after controlling for other risk factors, a participant with LDL-C PGS in Q1 would have seen a 3.82 mg/dL greater decrease in LDL-C in response to coaching than a participant with LDL-C PGS in Q2 or Q3. This suggests that a lifestyle coaching program may be more effective at lowering LDL-C for an individual with genetic predisposition for lower LDL-C relative to an individual with higher genetic risk. This result may not be surprising, as poor lifestyle choices may explain why someone with low genetic risk still has high LDL-C. These results are consistent with earlier studies showing high genetic predisposition for adverse lipid profiles limits the improvement in total cholesterol in response to lifestyle change^7,8^. Importantly, our results suggest that as the understanding of genetic predisposition continues to improve, so too will the ability to provide targeted personalized lifestyle recommendations, as well as the ability to identify when medical treatment is the best course of action.

This study has several limitations. As an observational study without a control group, we cannot separate the effect of coaching and the effect of being provided personalized data. In the future, it would be interesting to compare the full Scientific Wellness program to a standard coaching program that did not provide any clinical or genetic data to measure these effects separately. The lack of a control group may be particularly limiting for analyzing changes stratified by baseline clinical marker values, as regression to the mean could lead to biased estimates of effects^10^. We attempted to control for this by reporting changes in the total population as well as the out-of-range population. A pattern of improvements in the baseline OOR strata but worsening in the normal strata may be indicative of regression to the mean effects arising due to measurement variability.

Due to the personalized nature of the coaching, not all participants were working on improving all out of range clinical markers. Thus, our results may under-estimate the actual impact of health coaching on clinical outcomes. Coaches were aware of participants’ genetic predispositions when they generated personalized recommendations, which could lead to a bias in which participants with greater genetic predispositions (e.g. for higher LDL-C) received more aggressive lifestyle interventions. However, our results indicate that participants with greater genetic predispositions improve less in the program relative to participants with lower genetic predispositions. Therefore, any coaching bias to intervene more aggressively would act to attenuate our results rather than amplify them.

An additional potential limitation is the issue of compliance bias, which we were unable to address in the current study. Hypothetically, individuals who know they have higher - or lower - genetic risk for a trait may be less motivated to actively engage in lifestyle change. Some studies^3^ have reported greater self-reported behavior change in people who learned they were high-risk genetic carriers compared to low-risk non-carriers, but others^2,18^ did not find a relationship between genetic risk score and behavior change. Importantly, previous studies did not involve interactions with a trained lifestyle coach who can identify underlying core motivations and provide behavioral support and accountability to drive sustained behavior change. At the start of this study, data on participants’ actions and compliance were not collected in a way suitable for analysis; these data are now being collected and will be analyzed in the future.

Human wellness and disease are complex biological phenomena. The Scientific Wellness approach deals with this complexity by generating large amounts of multi-omic data, which we refer to as personal, dense, dynamic data (PD3) clouds, on many different biological systems for each individual. PD3 clouds can be used to understand an individual’s unique actionable possibilities for optimal wellness. This approach has the potential to transform our understanding of personalized medicine.

## Conclusions

This real-world study of a Scientific Wellness program demonstrated not only clinical improvements in participants with out of range biomarkers at baseline, but also many clinical improvements in the overall population, presumably related to sustained engagement and lifestyle changes. Furthermore, we report that genetic predisposition for nutrition and wellness-related phenotypes impacts clinical responses to a lifestyle coaching program. We believe that investigations into the relationship between genetic predispositions and the impact of lifestyle intervention will prove a fruitful avenue for further study.

## Methods

### Participants

All research was conducted in accordance to regulations and guidelines for observational research in human subjects. The study was reviewed and approved by the Western IRB (Study Number 1178906). The research was performed entirely using de-identified and aggregated data of individuals who had signed a research authorization allowing the use of their anonymized data in research. Per current U.S. regulations for use of deidentified data, informed consent was not required. To be eligible to join the program, participants had to be over 18 years of age, not pregnant, and a resident of any U.S. state except New York. The participants analyzed in this study are the 92% of participants who agreed to research use as of 6/19/2018 and enrolled in the program between July 2015 and March 2018.

### Personalized lifestyle coaching

Personalized, telephonic lifestyle coaching was provided to each participant in the program by registered dietitians, certified nutritionists, or registered nurses. A participant’s clinical data were available for them to view online via a data dashboard. To address specific OOR clinical markers, coaches provided lifestyle recommendations based on published scientific evidence which were further personalized in the context of the participant’s health goals and relevant genetic predispositions. Coaches did not make recommendations solely based on genetic risk, although they might take genetics into account when developing a behavioral plan for an out-of-range biomarker. For example, reducing sodium or caffeine might be recommended to any participant with high blood pressure, but if they also had risk alleles indicating enhanced susceptibility to dietary sodium or caffeine, this would be emphasized. See Supplementary Methods for details on personalized lifestyle coaching and Supplementary Table 4 for general clinical recommendations given for out-of-range biomarkers.

### Lab Data

Fasting blood draws were scheduled every 6 months but actual collection times varied. Salivary cortisol measurements were collected at home using a 4-time-point collection procedure and analyzed by ZRT (Beaverton, OR). Blood pressure measurements were recorded at each blood draw, and some participants provided additional self-reported measurements between visits via the data dashboard.

All laboratory tests were performed in CLIA-approved labs. The labs provided reference ranges for a majority of these clinical markers. Reference ranges for blood pressure were defined by U.S. public guidelines^19^. See Supplementary Methods for details on lab data collection.

### Anthropometric Data

Height, weight, and waist circumference were measured either at the blood draws (45%) or were self-reported via an online assessment or through the Fitbit Aria scale. Reference ranges for anthropometric data were defined by U.S. public health guidelines^20^.

### Genetic Data

Genetic data were collected using whole genome sequencing for 2,380 participants or SNP microarray genotyping for 151 participants. Curated genetic markers relevant to nutrition and wellness were reported to all participants as part of the program. These included SNPs previously associated with a nutrition or wellness-related phenotype (e.g. rs4588 with Vitamin D^21,22^ and rs174537 with omega-3 and omega-6 fatty acids^12,23^), and polygenic scores (PGSs for LDL-C, HDL-C, triglycerides, BMI, and waist circumference. Each of these PGSs was constructed using publicly available summary statistics from published Genome-Wide Association Studies (GWAS)^11,24,25^. See Supplementary Methods for details on genotype calling.

### Polygenic Score Creation

Briefly, the set of SNPs included in a PGS was determined as follows. The Benjamini-Hochberg^26^ procedure was applied to the p-values for all SNPs tested in the GWAS to account for multiple testing by controlling the false discovery rate (FDR) at a 5% level. This FDR filtered set of SNPs was then further pruned using linkage disequilibrium (LD): pairs of SNPs in close proximity capturing highly correlated information (r^2^ > 0.2) were identified, and the SNP with the smaller p-value in the pair was kept; this was repeated until all remaining SNPs were mutually uncorrelated (r^2^ < 0.2 for all pairs). The PGS for each individual was then calculated by summing up the published effect size for each selected SNP multiplied by the number of effect alleles the individual carried for that SNP, across all of the selected SNPs. Missing genotypes were mean imputed using the effect allele frequency. See Supplementary Table 5 for the list of variants in each polygenic score and their associated effect sizes. The homocysteine polygenic score was computed based on specific rules, which are provided in Supplementary Table 5.

### Data and Sample Filtering

To be included in the analysis of a clinical marker (labs or anthropometrics), a participant was required to have a baseline measurement within 30 days of their first blood draw, and at least one follow-up measurement between 90 days and 15 months later. Measurements collected more than 15 months after the participant’s baseline blood draw were excluded. Blood draws reported as non-fasting were excluded (1.7% of all blood draws). Lipid measurements were excluded for participants who reported taking cholesterol-lowering medication; diabetes markers were excluded for participants who reported taking blood sugar medication; blood pressure measurements were excluded for participants who reported taking blood pressure medication. Additionally, 17 participants who reported having type 1 diabetes were excluded from analyses of diabetes markers.

### Longitudinal Changes in Clinical Markers

Generalized linear mixed models (GLMMs) were used to estimate the average change in each clinical marker after 6 and 12 months in the program. The actual collection times of measurements varied from participant to participant. Therefore, rather than treating time in the program as a categorical variable with pre-specified collection points, linear regression splines were used to fit time as a continuous variable, allowing for differences in the trajectory of change of the clinical marker throughout the course of the program. To adjust for potential confounding effects, age at baseline, sex, enrollment channel, genetic ancestry, observation season, and observation vendor were included as fixed effects covariates in each GLMM.

### Longitudinal Changes Stratified by Baseline Range

We classified participants into strata by their baseline measurements: those with baseline measurements within the healthy range (as defined by clinical reference ranges) were classified as ‘normal’, below this range as ‘low’, and above this range as ‘high’. We estimated the change at 6 and 12 months for the average participant by baseline strata using GLMMs as described above, with the addition of an interaction term between a categorical variable for baseline strata and the linear regression spline for time in the program. Changes were not estimated for baseline strata containing less than 50 participants.

### Association of Genetic and Clinical Markers at Baseline

Linear regression models were used to estimate the % of variation explained by the genetic markers (SNPs or PGSs) provided to participants, as well as SNP genotype or PGS quartile effect sizes on baseline levels of the corresponding clinical markers. The same covariates included in the longitudinal change models were included in these regressions.

### Impact of Genetic Markers on Longitudinal Changes

Each genetic marker tested for association with a particular clinical marker at baseline was also tested for an effect on the longitudinal change of that clinical marker. Linear mixed models (LMMs) were used to identify interaction effects between different SNP genotypes or PGS quartiles and clinical marker changes, adjusting for baseline clinical marker values after the same amount of time in the program. Fixed effects covariates based on the same potential confounding variables as described for the longitudinal change models were used in these models.

See Supplementary Methods for details on regression models. P-values for all analyses were adjusted for the effects of multiple hypotheses testing using the Benjamini-Hochberg procedure^26^ (Supplementary Tables 2 and 3). All of the Results discussed in this study were significant after multiple hypothesis correction.

## Supplementary information

Supplementary Information

Supplementary Table 5

Supplementary Tables 1 2 3

## Data Availability

The multi-omic dataset will be made available through Arivale to qualified researchers under an agreement with Arivale that protects the privacy of the Arivale participants. Please contact data-access@arivale.com for more information and to apply to access the data.
