# Supplementary material for: Genetic Predisposition Impacts Clinical Changes in a Lifestyle Coaching Program
Source: Sci Rep. 2019 May 2;9:6805. doi: 10.1038/s41598-019-43058-0 (PMC6497671; doi:10.1038/s41598-019-43058-0)
Supplement: Supplementary file 1 — Supplementary Information [file 41598_2019_43058_MOESM1_ESM.docx]

**SUPPLEMENTARY INFORMATION: Genetic Predisposition Impacts Clinical Changes in a Lifestyle Coaching Program**

*Niha Zubair PhD, Matthew P Conomos PhD, Leroy Hood MD, PhD, Gilbert S Omenn MD, PhD, Nathan D Price PhD , Bonnie J Spring PhD, Andrew T Magis PhD, Jennifer C Lovejoy PhD*

**SUPPLEMENTARY METHODS**

*Personalized lifestyle coaching*.  Personalized lifestyle coaching was provided to each participant in the program by registered dietitians, certified nutritionists, or registered nurses. A participant’s clinical data were available for them to view online via a data dashboard. Typically, each participant worked with one coach throughout their time in the program. Coaching was delivered via telephone, email, and text message at a frequency agreed upon by the coach and participant. Generally, coaches spoke to participants once per month by phone and more frequently via email or text message.

The behavior change model used in the program is Social Cognitive Theory (SCT). ^1^ Coaches were also trained in a variety of other behavioral approaches including motivational interviewing, ^2^ Appreciative Inquiry, ^3^ cognitive behavioral strategies, ^4^ and mindfulness, ^5,6^ which they would use with participants as appropriate for their specific situation.

A participant’s clinical data were available for them to view online via a data dashboard. To address specific out of range (OOR) clinical markers, coaches provided education and lifestyle recommendations based on published scientific evidence and further personalized in the context of the participant’s health goals and relevant genetic predispositions. Some examples of general clinical recommendations are shown in Supplementary Table 4. However, this does not capture the personalization that occurred since each intervention was tailored such that each person had different “actionable recommendations.” For example, “reduce saturated fat” could lead to a recommendation to “implement meatless Monday”, “avoid adding coconut oil to coffee”, or “replace full-fat cheese with low-fat cheese” depending on the participant’s current diet and preferences. It was rare, but when there were conflicting recommendations for two biomarkers (e.g. one that would lead to a reduced fat recommendation and another that would lead to a reduced carbohydrate recommendation) coaches would use their clinical judgement, looking at the overall risk pattern in the biomarkers along with the participant’s goal and current lifestyle to determine the best approach. In some cases a hybrid approach could be adopted (e.g. a “low-fat Mediterranean diet”). Coaches did not make recommendations solely based on genetic risk, although they might take genetics into account when developing a behavioral plan for an out-of-range biomarker. For example, reducing sodium or caffeine might be recommended to any participant with high blood pressure, but if they also had risk alleles indicating enhanced susceptibility to dietary sodium or caffeine, this would be emphasized. Clinical oversight for the coaching program was provided by physicians.  An independent, external physician ordered all clinical tests from CLIA-certified vendors and performed the initial review to assess OOR results and recommend referral to the primary care provider if medical follow-up was indicated. Physicians were available to consult with coaches about any complex cases or specific questions.

*Lab Data Collection.* Blood samples were collected by trained phlebotomists at the LabCorp or Quest phlebotomy center most convenient to the participant’s location. Four days in advance of each blood draw, participants were asked to discontinue non-prescription medications, including acetaminophen, ibuprofen, and over-the-counter cold remedies; 24 hours in advance, participants were asked to avoid alcohol, vigorous exercise, and products containing aspartame or monosodium glutamate; 12 hours in advance, participants were asked to fast (no food or drink except water) until after the draw was completed.

Salivary cortisol measurements were collected at home using a 4 time-point salivary cortisol kit, shipped at room temperature to the testing laboratory, and analyzed by ZRT (Beaverton, OR) using standard clinical procedures.

*Genotype Calling*. Regardless of the genotyping method, DNA was extracted from whole blood samples using standard procedures at Covance (Redmond, WA). Whole genome sequencing was performed by Wuxi, Inc. (Shanghai, China) in a CLIA-certified laboratory using Illumina HiSeq X with sequencing mode PE-150 and target 30x coverage (Illumina Inc., San Diego, USA). BWA 0.7.12 was used for alignment of raw sequencing data to reference sequence hg19, and variant calling was performed using GATK HaplotypeCaller with GATK 3.3.0, including indel local realignment followed by base quality recalibration. SNP microarray genotyping was performed by Diagnomics (San Diego, USA) in a CLIA-certified laboratory using the Multi-Ethnic Global Screening Array (Illumina Inc., San Diego, USA). GenomeStudio v2.0 was used for genotype calling with the standard Illumina cluster file posted in August 2017. Genotype imputation was performed with minimac3 (v2.0.1), utilizing Eagle v2.3.2 for phasing and the Haplotype Reference Consortium (HRC) Release 1.1 reference panel.

*Longitudinal Analyses*. All longitudinal and genetic effects presented in this study were estimated using regression models; linear regression, linear mixed models (LMMs), and generalized linear mixed models (GLMMs) were all used for various analyses. Below, we define variables and terminology required to fully understand the analyses, followed by a more detailed description of the regression models used for each analysis presented in the manuscript.

*Definition of ‘Standard’ Covariates for Regression Models.*  To adjust for potential confounding effects, the non-time-varying covariates age at baseline (modeled as a quadratic function), sex, enrollment channel, and genetic ancestry, as well as the time-varying covariates observation season and observation vendor (when multiple vendors were used) were included as fixed effects in all regression models. In what follows, we refer to these as the ‘standard’ covariates.

Enrollment channel is a categorical variable representing 6 different cohorts of participants defined by the manner in which they entered the program. Genetic ancestry is represented by principal components (PCs) 1-7 from an analysis of 107,280 linkage disequilibrium pruned autosomal SNPs with minor allele frequency > 5% using the combined PC-AiR^7^ and PC-Relate^8^ approach as described by Conomos et al. ^9^ Observation season represents when the observation was collected and is a categorical variable defined as follows: December, January, and February are Winter; March, April, and May are Spring; June, July, and August are Summer, and September, October, and November are Autumn.

Observations for some clinical markers were collected from multiple sources/vendors: measurements of some clinical blood markers were collected from LabCorp and Quest; measurements of other clinical blood markers were only collected from LabCorp; measurements of blood pressure and waist circumference were collected from LabCorp, Quest, and self-report; measurements of weight and BMI were collected from LabCorp, Quest, self-report, and Fitbit Aria scales; measurements of salivary cortisol were collected only from ZRT. Observation vendor is a categorical variable representing the different possible vendors; this was only used when observations of a clinical marker were collected from multiple vendors.

*Heterogeneous Residual Variance.* For all LMMs used to analyze clinical markers measured on multiple vendors, potential differences in the variance between vendors were accounted for by allowing for heterogeneous residual variance by vendor (modeling a unique residual variance for each vendor).

*Linear Regression Spline for Time in the Program.* In each regression model with longitudinal data, time in the program was fit as a continuous variable using linear regression splines that allowed for differences in the trajectory of change of the clinical marker throughout the course of the program. For lab data (blood labs, blood pressure, and salivary cortisol), the regression spline for time in the program included knots at 0, 6, 9, 12, and 15 months; for anthropometric data (. BMI, weight, and waist circumference), the regression spline included an additional knot at 3 months, as there was sufficient follow-up data (due to the collection rate of these measurements) between 0 and 3 months to estimate the trajectory over that time.

*Transformations of Clinical Marker Data.* Prior to regression analysis, the highest and lowest 0.25% of clinical marker values were winsorized; specifically, the highest and lowest 0.25% of values were truncated to the values of the 99.75^th^ and 0.25^th^ percentiles of the distribution, respectively. This process eliminated a handful of extreme outliers that could exhibit extremely high leverage or lead to model convergence issues for GLMMs.

Blood mercury measurements have a highly skewed distribution and were therefore analyzed using a Gamma family (see below); however, the Gamma family cannot have outcomes with value 0, so measurements of 0ug/L were transformed to 0.5ug/L (half of the smallest observed non-zero value).

*Definition of Baseline Strata for BMI and Weight.* For stratified analysis of BMI and weight, baseline strata were based on the BMI categories underweight (< 18.5 kg/m^2^), normal (18.5 - 24.9 kg/m^2^), overweight (25.0 - 29.9 kg/m^2^), and obese (>= 30 kg/m^2^).

*Longitudinal Changes in Clinical Markers.* GLMMs were used to estimate average changes for each clinical marker. The outcome variable in a GLMM was the clinical marker of interest. Clinical markers with relatively symmetric distributions were analyzed using GLMMs with a Gaussian family and identity link (LMMs), while clinical markers with highly skewed distributions were analyzed using GLMMs with a Gamma family and identity link. The threshold in distribution skewness for classification as ‘relatively symmetric’ vs. ‘highly skewed’ was determined by inspecting diagnostic plots of residuals from LMMs run on every clinical marker. Fixed effects in each GLMM included the ‘standard’ covariates to adjust for potential confounding as well as the linear regression spline for time in the program to estimate longitudinal changes. Each GLMM also included random intercepts and random slopes for time in the program, allowing for participant differences in the intercept and the rate of change of the clinical marker over time. Using this mixed model framework, the model coefficients of the linear regression spline for time in the program provided the estimated change for the average individual at 6 and 12 months.

The addition of interaction terms between indicator variables for baseline stratum range (‘Normal’, ‘Low’, or ‘High’) and the linear regression spline for time in the program provided the estimated change for the average individual in each stratum at 6 and 12 months.

*Association of Genetic and Clinical Markers at Baseline.* Linear regression models were used to assess the relationship between genetic markers and baseline levels of the corresponding clinical markers. For each clinical-genetic marker pair, two linear regression models were fit, each using baseline values of the clinical marker as the outcome variable. The first regression included a categorical variable representing the different possible genotypic values as a predictor (genotypes for SNPs or quartiles for polygenic scores), as well as the ‘standard’ covariates. This regression was used to estimate the average effect of each genotype on baseline levels of the clinical marker. The second regression dropped the categorical variable representing the genotypic values. Using the sum of squares from both regression models, we computed the partial R^2^ value for the genetic marker, which can be interpreted as the proportion of variability in baseline values of the clinical marker explained by that genetic marker.

*Impact of Genetic Markers on Longitudinal Changes*. Each genetic marker tested for association with a particular clinical marker at baseline was also tested for an effect on the longitudinal change of that clinical marker. The effect of interest was the difference in longitudinal change in the clinical marker between genotypes, comparing participants with the same baseline clinical marker values after the same amount of time in the program. Because the genetic markers being tested were correlated with baseline values of the clinical markers, estimating this desired genetic effect required including the baseline value of the clinical marker as a covariate in the regression model, rather than as an observation of the outcome variable. LMMs with random intercepts were used, where the outcome variable was the difference in the clinical marker of interest at follow up from baseline, and the predictor of interest was the genotype. Fixed effects covariates included the non-time-varying ‘standard’ covariates, as well as season at both baseline and follow up observation, the linear spline for time in the program at follow up observation, and the baseline value of the clinical marker. When data from multiple vendors was analyzed, vendor at both baseline and follow up observation were also included as fixed effect covariates, and heterogeneous residual variance by vendor pair was modeled (e.g. Quest-Quest vs. Quest-LabCorp vs. LabCorp-LabCorp).

*Software.* Linear regression was run using the base lm() function in R, LMMs were run using the nlme package in R, and GLMMs were run using the glmmTMB package in R.

**SUPPLEMENTARY REFERENCES**

1. Bandura, A. Health promotion from the perspective of social cognitive theory. *Psychology and Health* **13,** 623–649 (2007).

2. Lundahl, B. W., Kunz, C., Brownell, C., Tollefson, D. & Burke, B. L. A Meta-Analysis of Motivational Interviewing: Twenty-Five Years of Empirical Studies:. *Research on Social Work Practice* **20,** 137–160 (2010).

3. Whitney, D. D. & Trosten-Bloom, A. *The Power of Appreciative Inquiry*. (Berrett-Koehler Publishers, 2010).

4. Butler, A., Chapman, J., Forman, E. & Beck, A. The empirical status of cognitive-behavioral therapy: A review of meta-analyses. *Clinical Psychology Review* **26,** 17–31 (2006).

5. Gu, J., Strauss, C., Bond, R. & Cavanagh, K. How do mindfulness-based cognitive therapy and mindfulness-based stress reduction improve mental health and wellbeing? A systematic review and meta-analysis of mediation studies. *Clinical Psychology Review* **37,** 1–12 (2015).

6. Grossman, P., Niemann, L., Schmidt, S. & Walach, H. Mindfulness-based stress reduction and health benefits. A meta-analysis. *J Psychosom Res* **57,** 35–43 (2004).

7. Conomos, M. P., Miller, M. B. & Thornton, T. A. Robust inference of population structure for ancestry prediction and correction of stratification in the presence of relatedness. *Genet. Epidemiol.* **39,** 276–293 (2015).

8. Conomos, M. P., Reiner, A. P., Weir, B. S. & Thornton, T. A. Model-free Estimation of Recent Genetic Relatedness. *Am. J. Hum. Genet.* **98,** 127–148 (2016).

9. Conomos, M. P. *et al.* Genetic Diversity and Association Studies in US Hispanic/Latino Populations: Applications in the Hispanic Community Health Study/Study of Latinos. *Am. J. Hum. Genet.* **98,** 165–184 (2016).

**SUPPLEMENTARY FIGURE TITLE AND LEGEND**


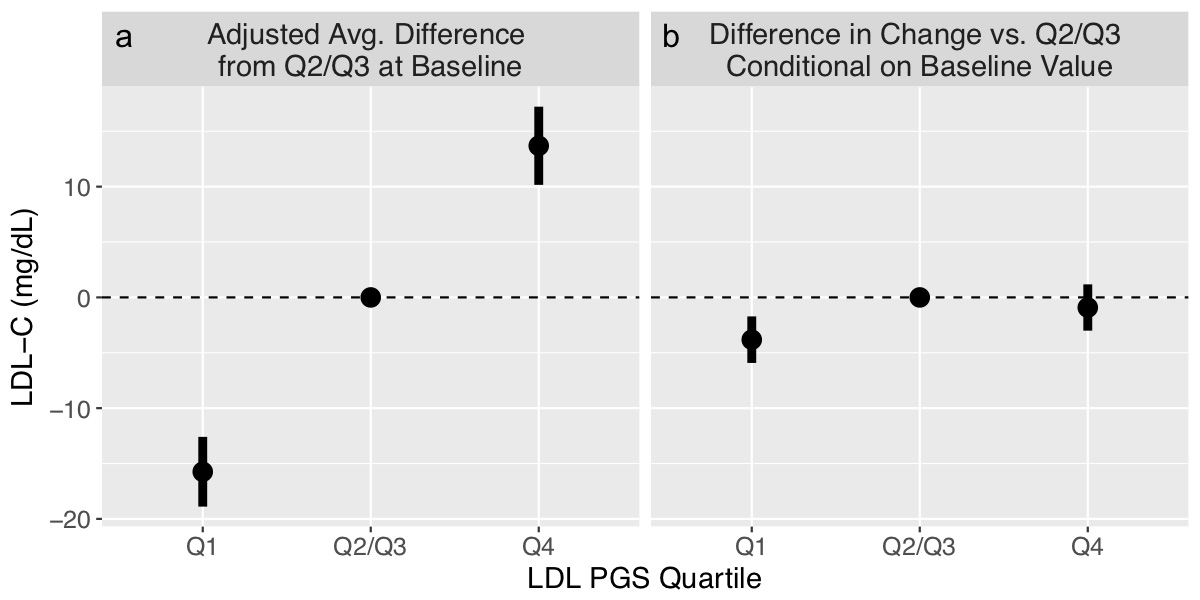


**Supplementary Figure 1. Effect of LDL-C polygenic score on baseline levels and longitudinal changes**

Panel a: Adjusted average difference in baseline LDL-C levels from those in LDL-C polygenic score (PGS) quartile 2 or 3 (Q2/Q3).

Panel b: Average difference in longitudinal change of LDL-C levels compared LDL-C PGS Q2 or Q3, conditional on baseline LDL-C and amount of time in the program.

In panels, the points represent the estimates from the linear regression models and LMMs, respectively, and the vertical bars show the 95% confidence intervals.

**TABLES AND FOOTNOTES**

**Supplementary Table 1: Clinical characteristics of the study population**

**Supplementary Table 2: Longitudinal changes from generalized linear mixed model analyses stratified by baseline range**

**Supplementary Table 3: Effects of genetics on baseline measures and on longitudinal changes of clinical markers**

**Supplementary Table 4 – Coaching recommendations for out of range biomarkers**

| **Biomarker** | **Examples of high-level coaching recommendations ^a^** |
| --- | --- |
| LDL cholesterol and other lipids | Reduce saturated fat intake, weight loss, increase fiber, increase exercise, increase dietary monounsaturated fats |
| Blood pressure | DASH diet, exercise, weight loss, manage stress |
| Homocysteine | Increase sources of methyl-folate, B-vitamins |
| HbA1c and other glycemic markers | Weight loss, increase exercise, reduce refined carbohydrates and sugar, reduce dietary fat |
| hsCRP and other inflammatory markers | Depends on cause of inflammation, if known. Anti-inflammatory diet strategies may be recommended. |
| Omega-3 fatty acids | Increase dietary omega-3 fatty acid intake, fish oil supplement |
| Vitamin D | Vitamin D supplement |
| Ferritin, hematocrit, hemoglobin | Iron supplement and/or dietary iron sources if low |
| Magnesium, zinc, MMA | Increase dietary intake or supplement if low |
| Salivary cortisol | Stress management techniques, improve sleep quality/quantity, stabilize blood sugar |

^a^ High-level recommendations were individually tailored and personalized by coaches to each participant’s lifestyle, goals and preferences as well as genetics when appropriate. In addition to lifestyle recommendations, when any value was out of range enough such that the ordering physician recommended medical follow up, coaches would include a referral to the member’s primary care physician.

**Supplementary Table 5 – List of variants and effect sizes for polygenic scores used in this study.**
